# 2-(Ammonio­meth­yl)pyridinium sulfate monohydrate

**DOI:** 10.1107/S1600536812007714

**Published:** 2012-02-29

**Authors:** M. Schutte, H. G. Visser, A. Roodt

**Affiliations:** a Department of Chemistry, University of the Free State, PO Box 339, Bloemfontein 9300, South Africa

## Abstract

In the crystal of the title hydrated molecular salt, C_6_H_10_N_2_^2+^·SO_4_^2−^·H_2_O, N—H⋯O and O—H⋯O hydrogen bonds link the mol­ecules into layers parallel to the *ab* plane. C—H⋯O hydrogen bonds are observed both within these layers and between mol­ecules and ions in adjacent layers.

## Related literature

For other salts of 2-amino­methyl­pyridine, see: Tooke *et al.* (2004 ▶); Mahjaub *et al.* (2005 ▶); Lemmerer *et al.* (2008 ▶); Khemiri *et al.* (2010 ▶); Døssing *et al.* (2001 ▶); Junk *et al.* (2006 ▶); Yuge *et al.* (2008 ▶).
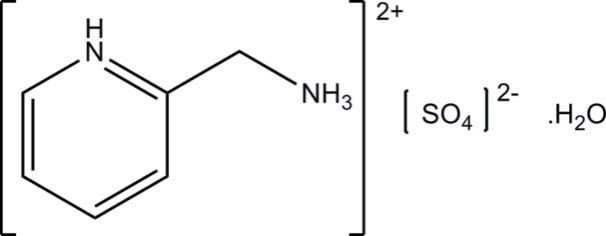


## Experimental

### 

#### Crystal data

C_6_H_10_N_2_^2+^·SO_4_^2−^·H_2_O
*M* *_r_* = 224.24
Triclinic, 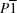

*a* = 5.2804 (1) Å
*b* = 6.9458 (2) Å
*c* = 12.4262 (3) Å
α = 81.392 (1)°
β = 82.874 (1)°
γ = 85.193 (1)°
*V* = 446.15 (2) Å^3^
*Z* = 2
Mo *K*α radiation
μ = 0.36 mm^−1^
*T* = 100 K
0.35 × 0.34 × 0.23 mm

#### Data collection

Bruker APEXII CCD diffractometer
Absorption correction: multi-scan (*SADABS*; Bruker, 2008 ▶) *T*_min_ = 0.881, *T*_max_ = 0.921
9334 measured reflections
1926 independent reflections
1846 reflections with *I* > 2σ(*I*)
*R*_int_ = 0.023

#### Refinement

*R*[*F* ^2^ > 2σ(*F* ^2^)] = 0.027
*wR*(*F* ^2^) = 0.075
*S* = 1.07
1926 reflections
151 parameters
H atoms treated by a mixture of independent and constrained refinement
Δρ_max_ = 0.35 e Å^−3^
Δρ_min_ = −0.49 e Å^−3^

### 

Data collection: *APEX2* (Bruker, 2008 ▶); cell refinement: *SAINT-Plus* (Bruker, 2008 ▶); data reduction: *SAINT-Plus*; program(s) used to solve structure: *SHELXS97* (Sheldrick, 2008 ▶); program(s) used to refine structure: *SHELXL97* (Sheldrick, 2008 ▶); molecular graphics: *DIAMOND* (Brandenburg & Putz, 2005 ▶); software used to prepare material for publication: *WinGX* (Farrugia, 1999 ▶).

## Supplementary Material

Crystal structure: contains datablock(s) global, I. DOI: 10.1107/S1600536812007714/fy2043sup1.cif

Structure factors: contains datablock(s) I. DOI: 10.1107/S1600536812007714/fy2043Isup2.hkl

Supplementary material file. DOI: 10.1107/S1600536812007714/fy2043Isup3.cml

Additional supplementary materials:  crystallographic information; 3D view; checkCIF report

## Figures and Tables

**Table 1:** Hydrogen-bond geometry (Å, °)

*D*—H⋯*A*	*D*—H	H⋯*A*	*D*⋯*A*	*D*—H⋯*A*
N2—H7*C*⋯O1^i^	0.87 (2)	2.587 (19)	2.8753 (14)	100.3 (14)
N2—H7*A*⋯O2^ii^	0.87 (2)	1.91 (2)	2.7514 (15)	161.2 (18)
N2—H7*B*⋯O4^iii^	0.875 (18)	1.944 (18)	2.8019 (15)	166.4 (16)
N2—H7*C*⋯O1^iv^	0.87 (2)	1.87 (2)	2.7320 (15)	168.0 (18)
N1—H8⋯O4	0.86 (2)	1.86 (2)	2.7170 (15)	173.0 (18)
O5—H9⋯O3	0.82 (2)	1.97 (3)	2.7928 (15)	173 (2)
O5—H10⋯O2^iii^	0.82 (3)	2.46 (2)	3.1822 (15)	149 (2)
O5—H10⋯O3^iii^	0.82 (3)	2.55 (3)	3.3009 (16)	154 (2)
C2—H2⋯O3^v^	0.93	2.39	3.2856 (16)	162
C3—H3⋯O5^vi^	0.93	2.57	3.2581 (17)	131
C6—H6*A*⋯O4	0.97	2.40	3.2080 (15)	141

Symmetry codes: (i) 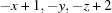; (ii) 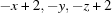; (iii) 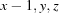; (iv) 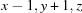; (v) 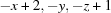; (vi) 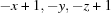.

**Fig. 1.**
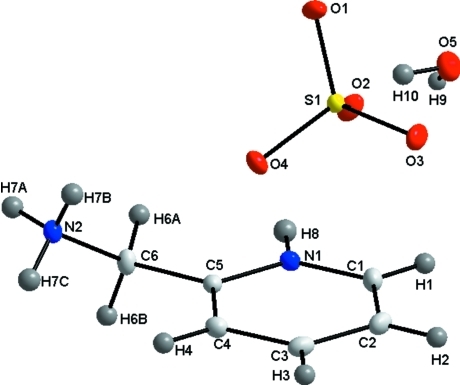
Diamond representation of the title compound, showing the numbering scheme and displacement ellipsoids (50% probability).

**Fig. 2.**
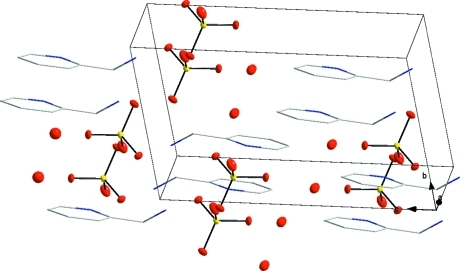
Packing of the title compound in the unit cell. Hydrogen atoms have been omitted for clarity.

## supplementary crystallographic information

### Comment

2-(Ammoniomethyl)pyridinium sulfate monohydrate crystallized in the triclinic spacegroup with both nitrogen atoms protonated in the 2-aminomethylpyridine molecule. One 2-(ammoniomethyl)pyridinium cation, one sulfate anion and one solvent water molecule are present in the asymmetric unit. The bond distances compare well with those of other similar reported structures (Tooke *et al.*, 2004; Mahjaub *et al.*, 2005; Døssing *et al.*, 2001; Junk *et al.*, 2006; Yuge *et al.*, 2008; Lemmerer *et al.*, 2008; Khemiri *et al.*, 2010). Also, the four sulfur-oxygen distances of 1.4694 (17) Å, 1.4718 (12) Å, 1.4741 (13) Å and 1.4965 (13) Å are well within range for sulfur-oxygen bond distances as well as the angles of 109.58 (7) °, 110.91 (7) °, 110.63 (7) °, 109.16 (8) °, 108.32 (6) ° and 108.18 (8) °. A total of eleven hydrogen bonds (N—H···O, O—H···O and C—H···O) are observed in the crystal structure. Seven of the hydrogen bonds are between the cations and sulfate anions, three are between a water molecule and sulfate anions and one is between the water molecule and the 2-(ammoniomethyl)pyridinium cation. The sulfate anions seems to surround the 2-(ammoniomethyl)pyridinium cation. The molecules pack in alternating layers parallel with the *ab* plane (Figure 2).

### Experimental

The crystal structure of (2-ammoniomethyl)pyridinium sulfate monohydrate was obtained by dissolving the ligand, 2-aminomethylpyridine, in water acidified with sulfuric acid. The final pH of the solution was recorded as pH = 6.1. The crystals were grown from this mixture by slow evaporation.

### Refinement

Aromatic H atoms were positioned geometrically and allowed to ride on their parent atoms, with *U*_iso_(H) = 1.2 *U*_eq_(parent) of the parent atom with a C—H distance of 0.93 Å. The methene H atoms were placed in geometrically idealized positions and constrained to ride on the parent atom with *U*_iso_(H) = 1.2 *U*_eq_(C) and at a distance of 0.97 Å. The O– and N– bound H atoms were placed from the electron density map and were refined freely with isotropic displacement parameters.

### Crystal data

**Table:**

C_6_H_10_N_2_^2+^·SO_4_^2^^−^·H_2_O	*Z* = 2
*M**_r_* = 224.24	*F*(000) = 236
Triclinic, *P*1	*D*_x_ = 1.669 Mg m^−^^3^
*a* = 5.2804 (1) Å	Mo *K*α radiation, λ = 0.71073 Å
*b* = 6.9458 (2) Å	Cell parameters from 7388 reflections
*c* = 12.4262 (3) Å	θ = 3.0–28.4°
α = 81.392 (1)°	µ = 0.36 mm^−^^1^
β = 82.874 (1)°	*T* = 100 K
γ = 85.193 (1)°	Cuboid, colourless
*V* = 446.15 (2) Å^3^	0.35 × 0.34 × 0.23 mm

### Data collection

**Table:**

Bruker APEXII CCD diffractometer	1846 reflections with *I* > 2σ(*I*)
Graphite monochromator	*R*_int_ = 0.023
φ and ω scans	θ_max_ = 27.0°, θ_min_ = 3.3°
Absorption correction: multi-scan (*SADABS*; Bruker, 2008)	*h* = −5→6
*T*_min_ = 0.881, *T*_max_ = 0.921	*k* = −8→8
9334 measured reflections	*l* = −15→15
1926 independent reflections	

### Refinement

**Table:**

Refinement on *F*^2^	Primary atom site location: structure-invariant direct methods
Least-squares matrix: full	Secondary atom site location: difference Fourier map
*R*[*F*^2^ > 2σ(*F*^2^)] = 0.027	Hydrogen site location: inferred from neighbouring sites
*wR*(*F*^2^) = 0.075	H atoms treated by a mixture of independent and constrained refinement
*S* = 1.07	*w* = 1/[σ^2^(*F*_o_^2^) + (0.0416*P*)^2^ + 0.2718*P*] where *P* = (*F*_o_^2^ + 2*F*_c_^2^)/3
1926 reflections	(Δ/σ)_max_ = 0.019
151 parameters	Δρ_max_ = 0.35 e Å^−^^3^
0 restraints	Δρ_min_ = −0.49 e Å^−^^3^

### Special details

**Table:**

Experimental. The intensity data were collected on a Bruker X8 ApexII 4 K Kappa CCD diffractometer using an exposure time of 10 s/frame. A total of 2250 frames were collected with a frame width of 0.5° covering up to θ = 28.43° with 97.3% completeness accomplished.
Geometry. All s.u.'s (except the s.u. in the dihedral angle between two l.s. planes) are estimated using the full covariance matrix. The cell s.u.'s are taken into account individually in the estimation of s.u.'s in distances, angles and torsion angles; correlations between s.u.'s in cell parameters are only used when they are defined by crystal symmetry. An approximate (isotropic) treatment of cell s.u.'s is used for estimating s.u.'s involving l.s. planes.
Refinement. Refinement of *F*^2^ against ALL reflections. The weighted *R*-factor *wR* and goodness of fit *S* are based on *F*^2^, conventional *R*-factors *R* are based on *F*, with *F* set to zero for negative *F*^2^. The threshold expression of *F*^2^ > 2σ(*F*^2^) is used only for calculating *R*-factors(gt) *etc*. and is not relevant to the choice of reflections for refinement. *R*-factors based on *F*^2^ are statistically about twice as large as those based on *F*, and *R*- factors based on ALL data will be even larger.

### Fractional atomic coordinates and isotropic or equivalent isotropic displacement parameters (Å^2^)

**Table:**

	*x*	*y*	*z*	*U*_iso_*/*U*_eq_	
S1	1.22129 (5)	−0.18907 (4)	0.83046 (2)	0.00951 (11)	
O4	1.07225 (18)	−0.01241 (13)	0.86655 (8)	0.0137 (2)	
O1	1.10127 (19)	−0.36349 (14)	0.89092 (8)	0.0170 (2)	
O3	1.2139 (2)	−0.18084 (14)	0.71186 (7)	0.0171 (2)	
O2	1.48757 (18)	−0.18785 (16)	0.85337 (8)	0.0206 (2)	
N1	0.7557 (2)	0.15757 (15)	0.71789 (9)	0.0113 (2)	
C1	0.7793 (3)	0.13486 (19)	0.61148 (11)	0.0144 (3)	
H1	0.9219	0.0649	0.5815	0.017*	
C2	0.5927 (3)	0.21501 (19)	0.54669 (11)	0.0148 (3)	
H2	0.608	0.2008	0.4729	0.018*	
C5	0.5565 (2)	0.25896 (18)	0.76561 (10)	0.0106 (2)	
C4	0.3629 (2)	0.34100 (18)	0.70404 (10)	0.0121 (3)	
H4	0.2225	0.4108	0.7358	0.015*	
C3	0.3814 (3)	0.31756 (18)	0.59413 (11)	0.0137 (3)	
H3	0.2518	0.3707	0.5522	0.016*	
C6	0.5689 (2)	0.27891 (19)	0.88357 (10)	0.0124 (3)	
H6A	0.6844	0.1755	0.9149	0.015*	
H6B	0.6376	0.4026	0.8875	0.015*	
O5	0.8069 (2)	−0.35284 (16)	0.64843 (9)	0.0215 (2)	
N2	0.3142 (2)	0.26919 (17)	0.94897 (9)	0.0116 (2)	
H9	0.924 (5)	−0.307 (3)	0.6720 (18)	0.034 (6)*	
H10	0.682 (5)	−0.305 (3)	0.6830 (19)	0.041 (6)*	
H7B	0.235 (3)	0.172 (3)	0.9344 (14)	0.014 (4)*	
H7C	0.226 (4)	0.380 (3)	0.9352 (15)	0.023 (4)*	
H7A	0.341 (4)	0.246 (3)	1.0179 (17)	0.023 (5)*	
H8	0.864 (4)	0.100 (3)	0.7607 (15)	0.021 (4)*	

### Atomic displacement parameters (Å^2^)

**Table:**

	*U*^11^	*U*^22^	*U*^33^	*U*^12^	*U*^13^	*U*^23^
S1	0.00975 (17)	0.00982 (17)	0.00827 (17)	0.00146 (11)	−0.00037 (11)	−0.00074 (11)
O4	0.0147 (4)	0.0112 (4)	0.0154 (4)	0.0017 (3)	−0.0013 (3)	−0.0048 (3)
O1	0.0185 (5)	0.0109 (4)	0.0181 (5)	0.0016 (4)	0.0055 (4)	0.0017 (4)
O3	0.0247 (5)	0.0183 (5)	0.0090 (5)	−0.0038 (4)	−0.0010 (4)	−0.0035 (4)
O2	0.0097 (5)	0.0313 (6)	0.0186 (5)	0.0013 (4)	−0.0033 (4)	0.0036 (4)
N1	0.0120 (5)	0.0098 (5)	0.0115 (5)	0.0013 (4)	−0.0014 (4)	−0.0009 (4)
C1	0.0165 (6)	0.0132 (6)	0.0128 (6)	−0.0004 (5)	0.0019 (5)	−0.0033 (5)
C2	0.0202 (6)	0.0143 (6)	0.0099 (6)	−0.0041 (5)	−0.0006 (5)	−0.0010 (5)
C5	0.0120 (6)	0.0088 (5)	0.0108 (6)	−0.0019 (4)	0.0005 (4)	−0.0015 (4)
C4	0.0116 (6)	0.0105 (6)	0.0140 (6)	−0.0006 (4)	−0.0008 (5)	−0.0012 (5)
C3	0.0153 (6)	0.0119 (6)	0.0138 (6)	−0.0039 (5)	−0.0042 (5)	0.0019 (5)
C6	0.0108 (6)	0.0154 (6)	0.0112 (6)	0.0005 (4)	−0.0007 (5)	−0.0039 (5)
O5	0.0183 (5)	0.0250 (5)	0.0229 (5)	−0.0029 (4)	−0.0016 (4)	−0.0087 (4)
N2	0.0119 (5)	0.0120 (5)	0.0108 (5)	0.0008 (4)	−0.0003 (4)	−0.0029 (4)

### Geometric parameters (Å, º)

**Table:**

S1—O2	1.4697 (10)	C5—C6	1.5028 (16)
S1—O3	1.4715 (9)	C4—C3	1.3900 (18)
S1—O1	1.4737 (10)	C4—H4	0.93
S1—O4	1.4971 (9)	C3—H3	0.93
N1—C5	1.3419 (16)	C6—N2	1.4835 (16)
N1—C1	1.3443 (17)	C6—H6A	0.97
N1—H8	0.86 (2)	C6—H6B	0.97
C1—C2	1.3783 (19)	O5—H9	0.82 (2)
C1—H1	0.93	O5—H10	0.82 (3)
C2—C3	1.3891 (19)	N2—H7B	0.875 (18)
C2—H2	0.93	N2—H7C	0.87 (2)
C5—C4	1.3860 (17)	N2—H7A	0.87 (2)
			
O2—S1—O3	109.59 (6)	C5—C4—H4	120.5
O2—S1—O1	110.90 (6)	C3—C4—H4	120.5
O3—S1—O1	110.65 (6)	C2—C3—C4	120.26 (12)
O2—S1—O4	109.13 (6)	C2—C3—H3	119.9
O3—S1—O4	108.33 (6)	C4—C3—H3	119.9
O1—S1—O4	108.17 (5)	N2—C6—C5	112.19 (10)
C5—N1—C1	122.78 (11)	N2—C6—H6A	109.2
C5—N1—H8	116.0 (12)	C5—C6—H6A	109.2
C1—N1—H8	121.1 (12)	N2—C6—H6B	109.2
N1—C1—C2	120.15 (12)	C5—C6—H6B	109.2
N1—C1—H1	119.9	H6A—C6—H6B	107.9
C2—C1—H1	119.9	H9—O5—H10	101 (2)
C1—C2—C3	118.50 (12)	C6—N2—H7B	110.1 (11)
C1—C2—H2	120.8	C6—N2—H7C	109.4 (13)
C3—C2—H2	120.8	H7B—N2—H7C	111.4 (17)
N1—C5—C4	119.20 (11)	C6—N2—H7A	106.9 (12)
N1—C5—C6	115.55 (11)	H7B—N2—H7A	108.3 (16)
C4—C5—C6	125.22 (11)	H7C—N2—H7A	110.7 (17)
C5—C4—C3	119.09 (12)		

### Hydrogen-bond geometry (Å, º)

**Table:**

*D*—H···*A*	*D*—H	H···*A*	*D*···*A*	*D*—H···*A*
N2—H7*C*···O1^i^	0.87 (2)	2.587 (19)	2.8753 (14)	100.3 (14)
N2—H7*A*···O2^ii^	0.87 (2)	1.91 (2)	2.7514 (15)	161.2 (18)
N2—H7*B*···O4^iii^	0.875 (18)	1.944 (18)	2.8019 (15)	166.4 (16)
N2—H7*C*···O1^iv^	0.87 (2)	1.87 (2)	2.7320 (15)	168.0 (18)
N1—H8···O4	0.86 (2)	1.86 (2)	2.7170 (15)	173.0 (18)
O5—H9···O3	0.82 (2)	1.97 (3)	2.7928 (15)	173 (2)
O5—H10···O2^iii^	0.82 (3)	2.46 (2)	3.1822 (15)	149 (2)
O5—H10···O3^iii^	0.82 (3)	2.55 (3)	3.3009 (16)	154 (2)
C2—H2···O3^v^	0.93	2.39	3.2856 (16)	162
C3—H3···O5^vi^	0.93	2.57	3.2581 (17)	131
C6—H6*A*···O4	0.97	2.4	3.2080 (15)	141

Symmetry codes: (i) −*x*+1, −*y*, −*z*+2; (ii) −*x*+2, −*y*, −*z*+2; (iii) *x*−1, *y*, *z*; (iv) *x*−1, *y*+1, *z*; (v) −*x*+2, −*y*, −*z*+1; (vi) −*x*+1, −*y*, −*z*+1.
